# miR-26a Inhibits Feline Herpesvirus 1 Replication by Targeting SOCS5 and Promoting Type I Interferon Signaling

**DOI:** 10.3390/v12010002

**Published:** 2019-12-18

**Authors:** Jikai Zhang, Zhijie Li, Jiapei Huang, Hang Yin, Jin Tian, Liandong Qu

**Affiliations:** Division of Zoonosis of Natural Foci, State Key Laboratory of Veterinary Biotechnology, Harbin Veterinary Research Institute, Chinese Academy of Agricultural Sciences, Harbin 150069, China; 15776628009@163.com (J.Z.); lizhijie.1982@126.com (Z.L.); sicaujiapeihuang@163.com (J.H.); yinhvri@163.com (H.Y.)

**Keywords:** feline herpesvirus 1, miR-26a, type I interferon, interferon stimulate genes, SOCS5, p-STAT1

## Abstract

In response to viral infection, host cells activate various antiviral responses to inhibit virus replication. While feline herpesvirus 1 (FHV-1) manipulates the host early innate immune response in many different ways, the host could activate the antiviral response to counteract it through some unknown mechanisms. MicroRNAs (miRNAs) which serve as a class of regulatory factors in the host, participate in the regulation of the host innate immune response against virus infection. In this study, we found that the expression levels of miR-26a were significantly upregulated upon FHV-1 infection. Furthermore, FHV-1 infection induced the expression of miR-26a via a cGAS-dependent pathway, and knockdown of cellular cGAS significantly blocked the expression of miR-26a induced by poly (dA:dT) or FHV-1 infection. Next, we investigated the biological function of miR-26a during viral infection. miR-26a was able to increase the phosphorylation of STAT1 and promote type I IFN signaling, thus inhibiting viral replication. The mechanism study showed that miR-26a directly targeted host SOCS5. Knockdown of SOCS5 increased the phosphorylation of STAT1 and enhanced the type I IFN-mediated antiviral response, and overexpression of suppressor of the cytokine signalling 5 (SOCS5) decreased the phosphorylation of STAT1 and inhibited the type I IFN-mediated antiviral response. Meanwhile, with the knockdown of SOCS5, the upregulated expression of phosphorylated STAT1 and the anti-virus effect induced by miR-26a were significantly inhibited. Taken together, our data demonstrated a new strategy of host miRNAs against FHV-1 infection by enhancing IFN antiviral signaling.

## 1. Introduction

FHV-1 is a member of the Varicellovirus genus of the subfamily Alphaherpesvirinae [1,2], which mainly infects domestic cats as well as other members of Felidae [3,4,5,6,7,8], and leads to feline viral rhinotracheitis. Like other herpesviruses, latent chronic infection is a characteristic of FHV-1 infection. FHV-1 can hide in the trigeminal ganglia of cats in latency and can reactivate under certain conditions, which is also a latent infectious source [9,10]. Previous studies indicated that α-herpesviruses usually encoded 65–80 open reading frames (ORFs) [11], and similarly, FHV-1 encoded 74 viral proteins [12]. These viral proteins contribute to manipulating the host innate immune response. Our previous studies found that a total of 11 viral proteins (UL30, ICP0, UL11, UL55, UL1, UL45, UL27, UL3.5, UL48, UL4 and US3) could inhibit the IFN-β promoter activity [13]. Activated type I IFN signalling is suppressed within 12 h after FHV-1 infection [13], but it is unclear how and what ways the host would use to counteract viral immune evasion.

MicroRNAs are short noncoding RNAs of approximately 20–24 nt that are processed by two RNase III enzymes (Drosha and Dicer) from long primary RNAs [14,15,16], which play a crucial part in the regulation of gene expression post-transcriptionally through mRNA degradation or translational inhibition via seed regions complementary to the 3′ UTR of target genes [17,18]. Many miRNAs have been demonstrated to play important regulatory roles in the innate immune response [19]. Some studies have demonstrated that JAK-STAT signalling could be regulated by host miRNAs by targeting the proteins of the suppressor of the cytokine signalling (SOCS) family, such as SOCS1, SOCS2, SOCS3 and SOCS5 [20]. miR-155 feedback enhanced type I IFN signalling by suppressing SOCS1 and inhibiting viral replication [21]. miR-30a-5p downregulated the expression of SOCS1 and SOCS3 via directly targeting their 3’ UTRs [22], which also enhanced IFN-I antiviral signalling. miR-130b and miR-432 enhanced the expression of IFN-β by targeting cellular SOCS5 [23,24]. During viral infection, host innate immunity is blocked at an early stage, and our previous studies also suggested that activated type I IFN signalling was quickly suppressed after FHV-1 infection [13], but some miRNAs are still upregulated to enhance IFN signalling pathways [21,23]. More importantly, it has been reported that some microRNAs can also inhibit virus replication by targeting viral genomes directly [25,26]. Given the critical roles of miRNAs in regulating type I IFN signalling, it is unknown whether the host uses these miRNAs to restart the IFN signalling pathways upon FHV-1 infection.

To explore the vital role of miRNAs involved in the process of host resistance to FHV-1 infection, small RNA high-throughput sequencing was performed following FHV-1 infection ([27] unpublished).

The results showed that miR-26a was significantly upregulated upon infection. In this study, we demonstrated that miR-26a could suppress FHV-1 replication by enhancing type I IFN-induced antiviral signalling by directly targeting a negative regulator of this pathway, SOCS5. FHV-1 infection induced the expression of miR-26a in a cGAS-dependent manner. Moreover, SOCS5 plays a vital role in the host response to FHV-1 infection, and knockdown of SOCS5 would reduce the antivirus effect induced by miR-26a. This study reveals a new strategy in which host miRNAs are involved in inhibiting FHV-1 replication by regulating the innate immune signalling pathway.

## 2. Materials and Methods

### 2.1. Cells and Virus

F81 (feline kidney cells) [28] and 293T cells were both purchased from ATCC and cultured in Dulbecco’s modified Eagle’s medium (DMEM; Gibco, Thermo Fisher Scientific, Waltham, MA, USA) supplemented with 10% foetal bovine serum (FBS; Excel, Australia) in a 37 °C incubator with 5% CO2. HR-1, an FHV-1 strain saved in our laboratory, was separated from a domestic cat in Harbin [13]. The titres of virus stock were measured by TCID50 assay. Multiplicity of infection (MOI) represents the number of viruses per cell (plaque-forming unit (PFU)/cell).

### 2.2. siRNAs, miRNAs and Transfection

siRNAs, miRNA mimics and inhibitors were synthesized as listed in Table S1 by Gene Pharma Company (Shanghai, China). Mimics are chemosynthetic double-strand RNAs that are similar to miRNA sequence, used for upregulating miRNA expression level; and inhibitors are chemosynthetic single-strand RNAs that are completely complementary to miRNA sequence, used for inhibiting endogenous miRNA expression level. Negative control (NC) mimics and inhibitors or siRNAs are chemosynthetic RNAs that have no effect on cellular miRNAs, serving as controls in the experiments. All siRNAs or miRNAs and plasmids were transfected with Opti-MEM (Gibco, USA) into cells using RNAiMAX (Invitrogen, Carlsbad, CA, USA) and Lipofectamine 2000 (Invitrogen, USA), respectively, according to the manufacturer’s protocol.

### 2.3. Antibodies and Western Blot

F81 cells were lysed on ice for 30 min using RIPA lysis buffer (moderate) (Beyotime, Shanghai China) supplemented with PMSF (Beyotime, China) and cocktail protease inhibitor II (MedChemExpress, MCE, Monmouth Junction, NJ, USA) before boiling with 5×SDS loading buffer for 10 min. Then, the proteins were separated by 10% SDS-PAGE gel and transferred onto BioTrace™ NT Nitrocellulose (NC) Transfer Membrane (Pall Life Sciences, New York, NY, USA). Next, the membrane was blocked using 5% skimmed milk for 1 h and incubated with primary antibodies as follows for 1 h at room temperature or overnight at 4 °C: rabbit anti-cGAS (ab176177), rabbit anti-p-STAT1 (phospho Y701) (Abcam, Cambridge, UK, ab109457, 1:1000), rabbit anti-STAT1 (Abcam, ab92506, 1:1000), rabbit anti-SOCS5 (Abcam, ab97283, 1:500), rabbit anti-GAPDH (Sigma-Aldrich, St. Louis, MI, USA, G9545, 1:3000), mouse anti-Flag (Sigma-Aldrich, F1804, 1:3000) and mouse anti-FHV-1 UL42 (1:200). After washing with TBST buffer, the membrane was incubated with IRDye^®^ 800CW goat anti-rabbit or anti-mouse secondary antibodies (LI-COR, Lincoln, NE, USA) for 1 h at room temperature. After washing again with TBST buffer, the proteins were detected using Odyssey CLx Image Studio 3.1 (LI-COR, USA). The anti-FHV-1 UL42 antibody was generated by immunizing six-week-old BALB/c mice with prokaryotically-expressed FHV-1 UL42 protein for three times at two weeks intervals. The serum of mice was served as polyclonal antibodies against FHV-1 UL42.

### 2.4. microRNA Target Gene Prediction and Plasmid Construction

RNA22 (https://cm.jefferson.edu/rna22/Interactive/) was used to predict the target genes of miR-26a in the FHV-1 genome. Additionally, the target genes of miR-26a in the host were predicted by Targetscan 7.1 (http://www.targetscan.org/vert_71/), and SOCS5 was selected as a potential target for further research. The 3′ UTR sequences of feline SOCS5 around the miR-26a target site were amplified and cloned into the pmirGLO luciferase reporter vector (Promega, Madison, WI, USA) using the ClonExpress II One Step Cloning Kit (Vazyme, Nanjing, China). The mutant SOCS5 plasmid was constructed by mutating seed regions in the 3′ UTR of SOCS5 using a homologous recombination kit (Vazyme, China). Likewise, the full-length ORF of feline SOCS5 (GenBank: 101080522) was also amplified and cloned into pCMV-3×flag using this method. All the primers used are listed in Table S2.

### 2.5. Quantitative Reverse Transcription-PCR (qRT-PCR)

Total RNA was extracted from F81 cells using the AxyPrepTM Multisource Total RNA Miniprep Kit (Axygen, Corning Inc., Corning, NY, USA) according to the manufacturer’s instructions. A total of 1 μg of RNA was synthesized into cDNA using the FastKing RT kit (with gDNase) (TIANGEN, Beijing, China). Then, real-time PCR was further utilized for gene expression analysis using SYBR Green Mix (Bioer, Zhejiang, China) with a JENA qTOWER 2.2 instrument (Germany) and was performed as follows: 95 °C for 1 min, followed by 40 cycles of three steps (95 °C for 15 s, 55 °C for 30 s and 72 °C for 15 s). For miRNA analysis, total RNA obtained via TRIzol reagent (Ambion, Austin, TX, USA) was reverse transcribed using AMV reverse transcriptase (Takara, Kusatsu, Japan) together with specific stem-loop RT primers. Then, the real-time PCR amplification procedure was 95 °C for 1 min, followed by 40 cycles of two steps (95 °C for 15 s, 60 °C for 30 s). All samples were carried out in triplicate on the same plate, and the 18S gene or U6 snRNA was utilized as the reference gene. The expression level of genes was calculated by normalizing to that of 18S or U6 using the comparative ΔΔCt method, and the values were expressed as 2-ΔΔCt. All primers used are listed in Table S2 and were synthesized by Comate Bioscience Company (Jilin, China).

### 2.6. Dual-Luciferase Reporter Assays

To verify miRNA target genes, the constructed plasmids pmirGLO-socs5-26a (WT) and pmirGLO-socs5-26a (Mut) were transfected into 293T cells with NC, miR-26a mimics for 36 h using Lipofectamine 2000. Then, the cells were lysed for testing luciferase activities using a Dual-Luciferase Reporter Assay Kit (Promega, Madison, WI, USA) according to the manufacturer’s instructions. The data were processed by normalizing firefly luciferase activities to Renilla luciferase activities. All samples were independently repeated at least three times on the plate.

### 2.7. Plaque Assays

To measure the effects of miR-26a on FHV-1 replication, F81 cells were inoculated with 10-fold diluted viral supernatants for 1 h at 37 °C, and then 2×DMEM and 2% low melting-point agarose were mixed in equal volume and added to the plates after discarding the virus suspensions. When the gels were solidified, the plates were cultured in a 37 °C incubator upside down for 3–5 days. The number of plaques was calculated after staining with crystal violet.

### 2.8. Statistical Analysis

All the experimental results were obtained from at least three independent experiments. The statistical significance between the two groups was assessed by GraphPad Prism 7.0 software using unpaired *t*-tests. Significant differences are indicated as follows: * *p* < 0.05, ** *p* < 0.01 and *** *p* < 0.001.

## 3. Results

### 3.1. FHV-1 Infection Increases the Expression of miR-26a

**Figure 1:**
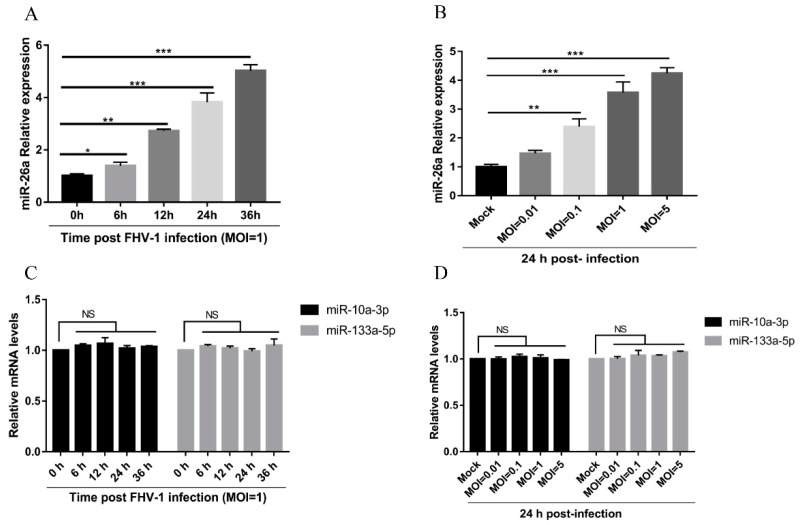
Feline herpesvirus 1 (FHV-1) infection increases miR-26a expression. (**A**,**B**) The miR-26a expression was measured in F81 cells infected with FHV-1 (MOI = 1) at the indicated time points (6, 12, 24, 36 h) (**A**) or with different multiplicity of infections MOIs (0.01, 0.1, 1, 5) at 24 hpi (**B**) by stem-loop qRT-PCR. (**C**,**D**) The miR-10a-3p and miR-133a-5p expression levels were measured in F81 cells infected with FHV-1 (MOI = 1) at the indicated time points (6, 12, 24, 36 h) (**C**) or at different MOIs ( 0.01, 0.1, 1, 5) at 24 hpi (**D**) by stem-loop qRT-PCR. The expression levels of various miRNAs were calculated by normalising to that of snRNA U6, and the uninfected groups served as the mock group. All samples were independently repeated three times, and data are representative of three independent experiments. The significant differences are indicated as follows: NS > 0.05, * *p* < 0.05, ** *p* < 0.01, *** *p* < 0.001.

High-throughput sequencing results have shown that miR-26a was upregulated after FHV-1 infection (Table S3). To investigate the biological function of miR-26a during viral infection, the expression level of miR-26a in F81 cells infected with FHV-1 was first evaluated using a stem-loop RT-qPCR method. Compared with the control group, miR-26a was significantly increased after FHV-1 infection at an MOI of 1 from 6 h to 36 h post-infection (Figure 1A). In addition, with the increase in viral inoculation dose, the expression levels of miR-26a displayed a gradually rising trend (Figure 1B). Both results demonstrate that miR-26a was upregulated with FHV-1 infection in a time- and MOI-dependent manner. We further analysed another two miRNAs, miR-10a-3p and miR-133a-5p, both of which were not affected upon infection as revealed by the high-throughput sequencing results. Results from the stem-loop RT-qPCR method showed that miR-10a-3p and miR-133a-5p were not significantly changed during the FHV-1 infection (Figure 1C,D). Therefore, FHV-1 infection results in the upregulation of miR-26a.

### 3.2. FHV-1 Infection Upregulates the Level of miR-26a via the cGAS-Mediated Signalling Pathway

**Figure 2:**
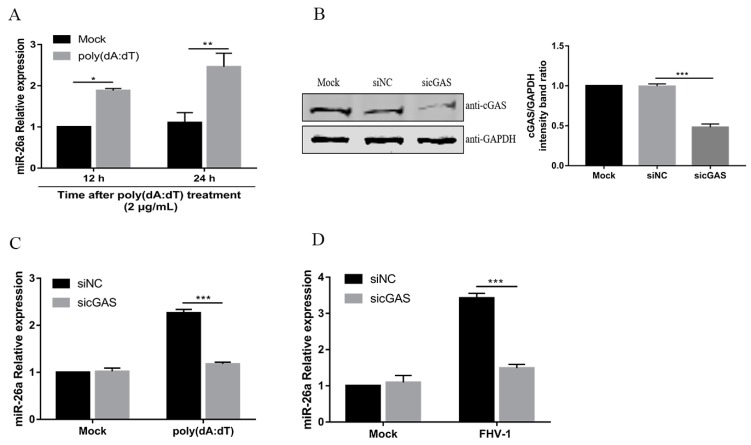
FHV-1 infection induces miR-26a expression via the cyclic GMP-AMP synthase (cGAS)-mediated signalling pathway. (**A**) miR-26a expression level was detected in cells transfected with 2 μg/mL poly (dA:dT) for 12 h or 24 h by stem-loop qRT-PCR. (**B**) The efficiency of cGAS knockdown was evaluated by western blotting (WB). (**C**,**D**) Knockdown of cGAS suppressed the expression of miR-26a induced by poly (dA:dT) or FHV-1 infection. F81 cells were transfected with sicGAS (60 nM) for 36 h followed by poly (dA:dT) treatment (2 μg/mL) (**C**) or FHV-1 infection (MOI = 1) (**D**) for another 24 h, and then cellular miR-26a was measured by stem-loop qRT-PCR. The expression level of miR-26a was calculated by normalizing to that of snRNA U6, and the uninfected groups served as the mock group. All samples were independently repeated three times, and data are representative of three independent experiments. The significant differences are indicated as follows: NS > 0.05, * *p* < 0.05, ** *p* < 0.01, *** *p* < 0.001.

A previous study showed that VSV and SeV induce miR-155 mainly through the retinoic acid-inducible gene 1 (RIG-I)-dependent pathway in macrophages [21]. RIG-I, as an RNA virus sensor, recognises viral double-stranded RNA to detect invading viruses [29]. Our previous study demonstrated that FHV-1 early infection could activate the DNA virus sensor, cyclic GMP-AMP synthase (cGAS), to induce the IFN-β [13]. Then, we investigated whether miR-26a was induced through the cGAS during FHV-1 infection. To confirm this, F81 cells were treated with poly(dA:dT), a synthetic double-stranded DNA, which can be sensed by the cGAS-STING pathway [30]. Then, the expression level of miR-26a was examined by qPCR. Indeed, miR-26a expression level was significantly increased after treatment with poly (dA:dT) for 12 h or 24 h (Figure 2A). To further examine the role of cGAS in the expression of miR-26a, endogenous cGAS was knocked down by the siRNA method (Figure 2B) and then FHV-1- or poly (dA:dT)- induced miR-26a expression level was analysed by qPCR. The results showed that knockdown of cGAS impaired miR-26a expression upon FHV-1 infection (Figure 2C) or poly (dA:dT) treatment (Figure 2D) and led to approximately 50% less expression than the mock transfection group. These data suggested that miR-26a was induced after FHV-1 infection through the cGAS-mediated signalling pathway.

### 3.3. miR-26a Inhibits FHV-1 Replication

**Figure 3:**
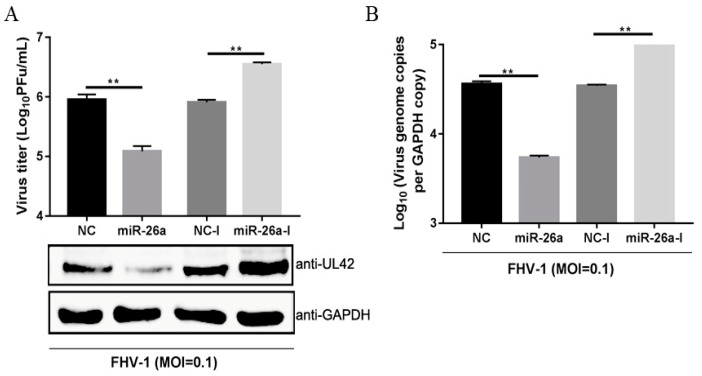
miR-26a suppresses FHV-1 replication. miR-26a mimics (80 nM) or inhibitor (160 nM) were transfected into F81 cells for 24 h, followed by FHV-1 infection at an MOI of 0.1 for 24 h. Then, the supernatants were collected for measuring virus titres via the virus plaque assay (**A**), and cell pellets were used to analyze FHV-1 protein expression (**A**) by WB or detect viral DNA copies (**B**) by absolute quantification PCR, respectively. GAPDH was used as an internal control in qPCR, and NC mimics (NC) and NC inhibitors (NC-I) served as negative controls. All samples were independently repeated three times, and data are representative of three independent experiments. The significant differences are indicated as follows: NS > 0.05, ** *p* < 0.01.

Due to the significant changes after FHV-1 infection, we examined whether miR-26a may be involved in FHV-1 replication. To explore the role of miR-26a in FHV-1 replication, F81 cells were transfected with miR-26a mimics or inhibitors for 24 h and inoculated with FHV-1 at an MOI of 0.1. At 24 hpi, the supernatant was harvested for the plaque assay, and the protein and RNA levels from cells were analysed through WB and qPCR, respectively. UL42, a DNA polymerase subunit of FHV-1, is an essential gene for viral replication, which can reflect the replication level of FHV-1, therefore, it was selected to detect FHV-1 replication level in the cells. As shown in Figure 3A, compared with the NC (negative control) mimics transfection group, transfection with miR-26a mimics (miR-26a) significantly reduced the expression of viral UL42 (Figure 3A Down) and resulted in a 10-fold decrease in virus production (Figure 3A Up), as well as a 7-fold decrease in DNA abundance (Figure 3B). Moreover, miR-26a inhibitors (miR-26a-I) exhibited significant promoting effects on viral protein expression and virus titres, as well as genome abundance relative to NC inhibitors (NC-I) (Figure 3A,B). All these data demonstrated that miR-26a could significantly suppress FHV-1 replication.

### 3.4. miR-26a Directly Targets SOCS5

**Figure 4:**
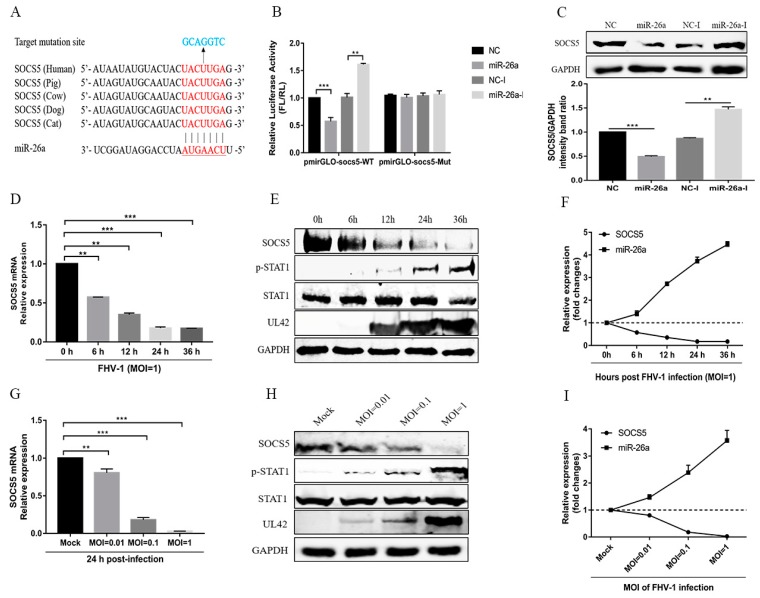
SOCS5 is a target gene of miR-26a. (**A**) Diagram of the predicted target sites of miR-26a in the 3′ UTR of SOCS5. The target sites of SOCS5 are indicated with red font, the mutant target sites of SOCS5 are indicated with blue font, and the seed regions of miR-26a are underlined. (**B**) Verification of target of miR-26a via the dual-luciferase assay. pmiRGLO-SOCS5-26a wild-type or mutant luciferase reporter plasmids were transfected into F81 cells, together with 80 nM miR-26a mimics or 160 nM miR-26a inhibitors. Thirty-six hours post-transfection, relative luciferase activities were tested and calculated by normalizing firefly luciferase activities to Renilla luciferase activities (FL/RL). (**C**) Verification of target of miR-26a via western blot. F81 cells were transfected with miR-26a mimics (80 nM) or inhibitors (160 nM). Forty-eight hours post-transfection, the cells were lysed for western blot analysis to test the expression of endogenous SOCS5. GAPDH served as an internal control. NC mimics and NC-I served as negative controls. (**D**–**F**) F81 cells were inoculated with FHV-1 at an MOI of 1, and then the mRNA (**D**) or protein (**E**) levels of SOCS5 as well as the level of p-STAT1 were analyzed by qRT-PCR or WB at the indicated time points (6, 12, 24, 36 h), respectively. The kinetic expression profile of SOCS5 and miR-26a with FHV-1 infection at different time points are shown in (**F**). (**G**–**I**) F81 cells were inoculated with FHV-1 at different MOIs (0.01, 0.1, 1), and the mRNA (**G**) or protein (**H**) levels of SOCS5, as well as the level of p-STAT1, were analysed by qRT-PCR or WB at 24 hpi, respectively. The kinetic expression profile of SOCS5 and miR-26a with FHV-1 infection at different MOIs are shown in (**I**). All samples were independently repeated three times, and data are representative of three independent experiments. The significant differences are indicated as follows: NS > 0.05, ** *p* < 0.01, *** *p* < 0.001.

Previous studies have reported that some miRNAs may inhibit virus replication by directly targeting viral genome [23], so, to further elucidate the underlying mechanism of miR-26a attenuating FHV-1 replication, we first analysed miR-26a targets within both FHV-1 sense and antisense RNA sequences using RNA22 (https://cm.jefferson.edu/rna22/Interactive/), but found no potential target sites in FHV-1 RNA. Then, the potential targets of miR-26a in the host were predicted using Targetscan software (http://www.targetscan.org/vert_71/). Computational analysis showed that the 3′ UTR of feline SOCS5, a negative regulatory factor for the JAK-STAT pathway, contains potential target sites of miR-26a, which is complementary to the seed region of this miRNA and conserved in other mammals (Figure 4A). To verify whether miR-26a directly targets SOCS5, the 3′ UTR of the target sites and its mutants were cloned into the luciferase reporter vector pmiRGLO. Dual-luciferase reporter assays indicated that miR-26a mimics significantly decreased luciferase activity by nearly 50%, while miR-26a inhibitors markedly increased that in the cells transfected with pmiRGLO-SOCS5 (Figure 4B Left). In contrast, miR-26a mimics and its inhibitors did not affect luciferase activity in the cells transfected with pmiRGLO-SOCS5 mutants (Figure 4B Right). These data suggest that SOCS5 may be a target gene of miR-26a. To further verify the results, we examined the protein levels of cellular SOCS5 after transfecting miR-26a mimics or its inhibitor. Overexpression of miR-26a significantly decreased the expression of endogenous SOCS5 in F81 cells, whereas the inhibition of miR-26a increased SOCS5 expression (Figure 4C). All these data demonstrate that SOCS5 is a target gene of miR-26a.

Since FHV-1 infection induced the expression of miR-26a, and miR-26a directly targets SOCS5, SOCS5 expression should be decreased and p-STAT1 expression should be increased after FHV-1 infection. Therefore, we examined the mRNA and protein expression levels of SOCS5 in F81 cells following with FHV-1 infection at different time points or different MOIs. As shown in Figure 4D,E, after FHV-1 infection at an MOI of 1, a significant reduction in the mRNA and protein levels of SOCS5 and a marked increase of p-STAT1 expression level was observed from 6 hpi to 36 hpi. Besides, FHV-1-mediated inhibition of SOCS5 mRNA and protein also displayed an MOI-dependent manner (Figure 4G,H). In combination with the results above (Figure 1A,B), SOCS5 showed a negative correlation with the kinetic expression profiles of miR-26a at different time points post-infection (Figure 4F) and different MOIs (Figure 4I). These data further suggested that FHV-1 infection induces the expression of miR-26a, which decreased the expression of SOCS5.

### 3.5. miR-26a Enhance JAK-STAT Antiviral Signalling

**Figure 5:**
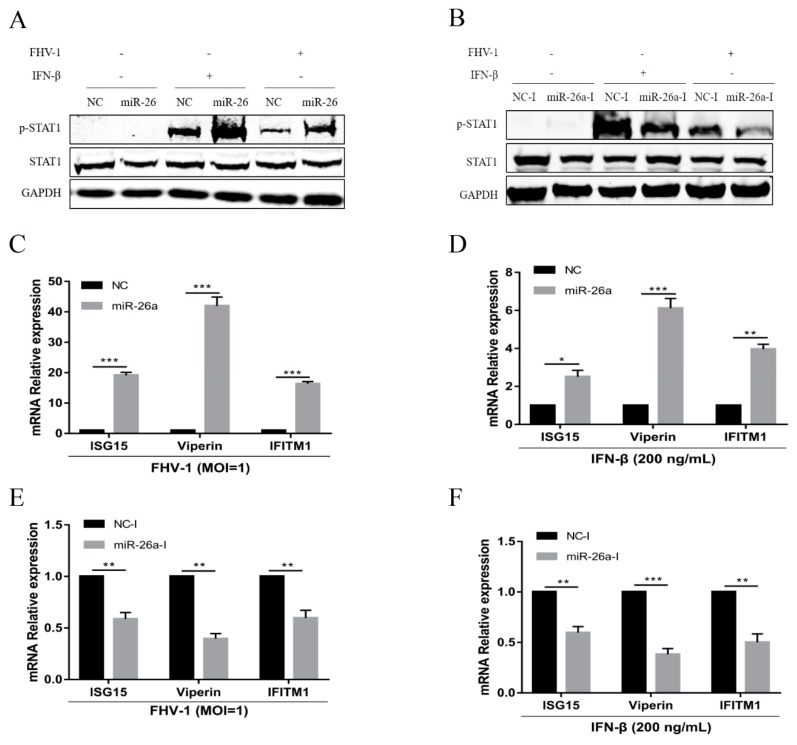
miR-26a enhances IFN-I-induced antiviral signalling. (**A**,**B**) miR-26a enhanced STAT1 phosphorylation level (p-STAT1), while blocking cellular miR-26a by inhibitors suppressed p-STAT1 expression level upon FHV-1 infection or IFN-β treatment. F81 cells were transfected with miR-26a mimics (80 nM) (**A**) or inhibitor (160 nM) (**B**) for 24 h and then inoculated with FHV-1 (MOI = 1) for 24 h or IFN-β stimulation (200 ng/mL) for 30 min, respectively. Then, the expression levels of p-STAT1 and STAT1 were identified by WB. GAPDH served as an internal control. (**C**–**F**) miR-26a enhanced the expression of downstream ISGs, while miR-26a inhibitors suppressed the production of ISGs induced by FHV-1 infection or IFN-β stimulation. F81 cells were transfected with miR-26a mimics (80 nM) (**C**,**D**) or inhibitors (160 nM) (**E**,**F**) for 24 h and then inoculated with FHV-1 (MOI = 1) for 24 h (**C**,**E**) or stimulated with IFN-β (200 ng/mL) for 12 h (**D**,**F**). Then, total RNAs were extracted for qRT-PCR to test the expression of ISG15, Viperin and IFITM1. The relative expression of these genes was calculated by normalising to that of cellular 18S RNA. NC mimics and NC inhibitors served as negative controls. All samples were independently repeated three times, and data are representative of three independent experiments. The significant differences are indicated as follows: NS > 0.05, * *p* < 0.05, ** *p* < 0.01, *** *p* < 0.001.

Activated JAK-STAT signalling induces hundreds of ISGs to inhibit viral infection, which is a common target regulated by host miRNAs through inducing the degradation of the suppressor of the cytokine signalling (SOCS) family [20]. SOCS5, serving as a target of miR-26a, is a negative regulator of JAK-STAT. So, to explore the effect of miR-26a on IFN antiviral signalling cascades, we analysed the level of phosphorylation of STAT1 (p-STAT1) (Y701) by WB assay. F81 cells were transfected with miR-26a mimics or inhibitors for 24 h, and the p-STAT1 expression levels were detected in the cells infected with FHV-1 for 24 h or stimulated with IFN-β for 30 min. With FHV-1 infection or IFN-β stimulation, the expression levels of p-STAT1 were significantly enhanced in the cells transfected with miR-26a mimics compared with levels in the NC mimics (Figure 5A), whereas the inhibition of endogenous miR-26a by inhibitor resulted in a decrease in p-STAT1s level (Figure 5B). Furthermore, we also measured the expression levels of three antiviral ISGs, including ISG15, Viperin and IFITM1, in the presence of miR-26a mimics or its inhibitor upon FHV-1 infection or IFN-β stimulation. Consistent with the p-STAT1 detection results, overexpression of miR-26a significantly upregulated the expression of these ISGs induced by FHV-1 infection (Figure 5C) or IFN-β stimulation (Figure 5D), and miR-26a inhibitors decreased the production of these ISGs (Figure 5E,F). All of these results demonstrated that miR-26a could promote type I IFN signalling.

### 3.6. miR-26a Is Involved in Type I Interferon Production

**Figure 6:**
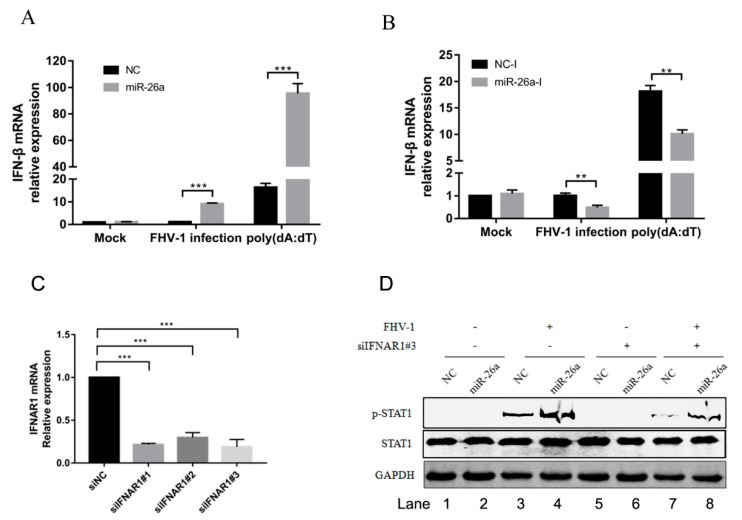
miR-26a promotes the expression of type I interferon. (**A**,**B**) miR-26a enhanced FHV-1 or poly (dA:dT)-induced expression of IFN-β, while miR-26a inhibitors impaired IFN-β expression level induced by FHV-1 infection or poly (dA:dT) treatment. miR-26a mimics (**A**) or inhibitors (**B**) were transfected into F81 cells at a concentration of 80 nM or 160 nM for 24 h, respectively, then followed by FHV-1 infection at an MOI of 1 or transfection of poly (dA:dT) at a concentration of 1 μg/mL. Twenty-four hours after FHV-1 infection or poly (dA:dT) treatment, total RNAs were extracted for qRT-PCR to measure the mRNA expression level of IFN-β. The relative expression of IFN-β was normalized to that of cellular 18S RNA, and NC mimics and NC inhibitors served as negative controls. (**C**) The efficiency of IFNAR1 knockdown was evaluated by qRT-PCR. Three siRNAs targeting on feline IFNAR1 were transfected into F81 cells at a concentration of 60 nM, respectively. 36 h after transfection, cellular IFNAR1 mRNA expression level was tested by qRT-PCR. (**D**) F81 cells were co-transfected with siIFNAR1#3 (60 nM) and miR-26a mimics (80 nM) for 24 h followed by FHV-1 infection (MOI = 1) for another 24 h, and then the expression levels of p-STAT1 and STAT1 were identified by WB. All samples were independently repeated three times, and data are representative of three independent experiments. The significant differences are indicated as follows: NS > 0.05, ** *p* < 0.01, *** *p* < 0.001.

ISGs induced by initial stimulation would form a positive feedback loop, which increases the production of IFN-β. To analyse whether the upregulated expression of these ISGs by miR-26a will increase the production of type I interferon, we transfected miR-26a mimics into F81 cells to examine the mRNA levels of IFN-β following with FHV-1 infection or poly (dA:dT) stimulation. As a result, overexpression of miR-26a significantly increased the expression of IFN-β in cells with FHV-1 infection (approximately 10-fold) or poly (dA:dT) treatment (approximately 5-fold) (Figure 6A). To further confirm this result, miR-26a inhibitors were transfected into F81 cells to evaluate the expression of IFN-β. In contrast, the production of IFN-β was markedly decreased to nearly 50% after FHV-1 infection or poly (dA:dT) treatment in comparison with that of NC inhibitors (Figure 6B). These data reveal that miR-26a is able to increase IFN-β expression in response to FHV-1 infection.

But the upregulation of p-STAT1 by miR-26a may be caused by the increase of IFN-β production, which further promoted the phosphorylation of STAT1. It was necessary to exclude that miR-26a promotes the phosphorylation of STAT1 by upregulating the expression of IFN-α/β. First, we screened an effective siRNA#3 targeting on IFNAR1 (Figure 6C), then analysed p-STAT1 level induced by miR-26a in the condition of IFNAR1 knockdown. Compared with the siNC control (Figure 6D Lane 3), knockdown of the IFNAR1 inhibited the level of p-STAT1 (Figure 6D Lane 7) upon FHV-1 infection, which demonstrated that knockdown of IFNAR1 blocked activation of downstream STAT1. However, compared with the NC mimics control (Figure 6D Lane 7), overexpression of miR-26a mimics could still promote the expression of p-STAT1 (Figure 6D Lane 8) upon virus infection, revealing that knockdown of IFNAR1 did not block the upregulated expression of p-STAT1 induced by miR-26a. So, the upregulated expression of p-STAT1 induced by miR-26a does not depend on the upstream IFN-α/β.

### 3.7. SOCS5 Can Inhibit Type I Antiviral Signalling and Facilitate FHV-1 Replication

**Figure 7:**
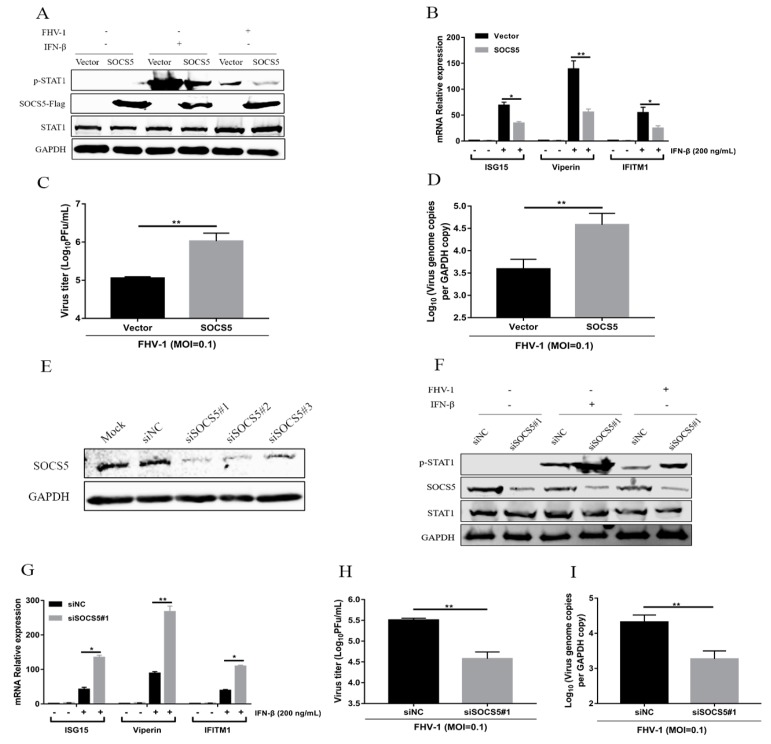
SOCS5 negatively regulates type I IFN signalling and facilitates FHV-1 replication. (**A**–**D**) Overexpression of SOCS5 blocked IFN- induced signalling cascades and promoted FHV-1 replication. F81 cells were transfected with p3×Flag-CMV-SOCS5 or empty vectors for 24 h, followed by FHV-1 infection (MOI = 1 for p-STAT1 expression and ISGs mRNA analysis, and MOI = 0.1 for virus titres analysis) for 24 h or IFN-β stimulation (200 ng/mL) for 30 min. Then, the expression levels of p-STAT1 and STAT1 were identified by WB analysis (**A**). GAPDH served as an internal control. Total RNA from another sample was extracted for qRT-PCR to detect the expression of ISG15, Viperin and IFITM1 (**B**). The relative expression levels of these genes were calculated by normalising to that of cellular 18S RNA. The supernatants and cells were collected for testing virus titres via the plaque assay (**C**) and virus copies by absolute quantification PCR (**D**), respectively. (**E**–**I**) Knockdown of endogenous SOCS5 enhanced IFN-elicited signalling cascades and suppressed FHV-1 replication. F81 cells were transfected with three siRNAs targeting SOCS5 for 48 h. The silencing efficiency was evaluated through testing cellular SOCS5 by western blot (**E**). siSOCS5#1 was selected for transfection into F81 cells. Thirty-six hours after transfection, the cells were infected with FHV-1 (MOI = 1 for p-STAT1 expression and ISGs mRNA analysis, and MOI = 0.1 for virus titres analysis) for 24 h or stimulated by IFN-β (200 ng/mL) for 30 min. Then, the protein levels of p-STAT1 (**F**) and the mRNA expression of ISG15, Viperin and IFITM1 (**G**) were detected according to the method described above. Virus titres (**H**) and virus copies (**I**) were determined via the plaque assay or absolute quantification PCR, respectively. Empty vectors and siNC groups served as negative controls. All samples were independently repeated three times, and data are representative of three independent experiments. The significant differences are indicated as follows: NS > 0.05, * *p* < 0.05, ** *p* < 0.01.

SOCS5 is a target of miR-26a, which inhibits FHV-1 infection via enhancing IFN signalling. To identify the role of SOCS5 in regulating JAK-STAT and anti-FHV-1 replication, SOCS5 was overexpressed for 24 h, and then the p-STAT1 level was examined after stimulation with FHV-1 infection or IFN-β treatment. SOCS5 overexpression significantly decreased the level of p-STAT1 upon both treatments (Figure 7A). Furthermore, SOCS5 overexpression also significantly downregulated the expression of three ISGs (ISG15, Viperin and IFITM1) by IFN-β treatment, compared with the vector control (Figure 7B). Then, due to the inhibition of JAK-STAT signalling by SOCS5, virus titres (Figure 7C) and genome copies (Figure 7D) of FHV-1 were further analysed in SOCS5 overexpressed cells by quantification PCR and plaque assays, respectively. As shown in Figure 7C,D, SOCS5 overexpression significantly promoted viral replication.

To further verify the function of SOCS5, we downregulated the expression of endogenous SOCS5 using RNA interference method. Evaluation of the knockdown efficiency of three siRNAs targeting SOCS5 was performed, which revealed that siSOCS5#1 showed the highest inhibition efficiency (Figure 7E), and was selected for the following experiments. F81 cells were transfected with siSOCS5#1 for 24 h and then inoculated with FHV-1 or IFN-β. Knockdown of endogenous SOCS5 enhanced the level of p-STAT1 upon FHV-1 infection or IFN-β stimulation (Figure 7F). Furthermore, knockdown of endogenous SOCS5 also significantly upregulated the expression of three ISGs (ISG15, Viperin and IFITM1) induced by the IFN-β treatment compared with the NC control (Figure 7G). Moreover, virus titres (Figure 7H) and genome copies (Figure 7I) were both decreased due to the knockdown of endogenous SOCS5. These data revealed that SOCS5 negatively regulates the JAK-STAT pathway and promotes FHV-1 replication.

### 3.8. miR-26a Enhances IFN Antiviral Signalling Through Regulating Downstream SOCS5

**Figure 8:**
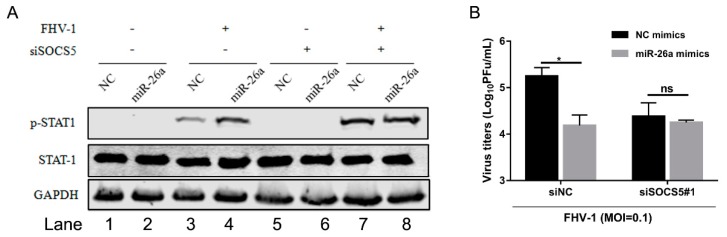
miR-26a enhances IFN antiviral signalling by regulating downstream SOCS5 instead of interferon. F81 cells were co-transfected with miR-26a mimics together with siSOCS5#1 for 24 h and followed by FHV-1 infection (MOI = 1 for p-STAT1 expression analysis and MOI = 0.1 for virus titres analysis). At 24 hpi, p-STAT1 levels were detected by WB (**A**) and virus titres in supernatants were measured via plaque assay (**B**). All samples were independently repeated three times, and data are representative of three independent experiments. The significant differences are indicated as follows: NS > 0.05, * *p* < 0.05.

To further verify that miR-26a regulated IFN antiviral signalling pathway by the SOCS5-STAT1 axis, we examined the level of p-STAT1 induced by miR-26a upon FHV-1 infection together with knockdown of SOCS5. miR-26a mimics were co-transfected into F81 cells together with siSOCS5#1 for 24 h, followed by FHV-1 infection for 24 h. Then, the expression level of p-STAT1, as well as virus titres, were examined. In the siNC-transfected group (Figure 8A Lane 1–4), miR-26a significantly enhanced p-STAT1 expression level (Figure 8A Lane 4) compared to the NC group (Figure 8A Lane 3) after FHV-1 infection. However, once SOCS5 expression level was knocked down, the levels of p-STAT1 between NC (Figure 8A Lane 7) and miR-26a (Figure 8A Lane 8) during FHV-1 infection were comparable, and miR-26a-mediated upregulation of p-STAT1 was inhibited compared to the NC group. On the other hand, miR-26a overexpression inhibited FHV-1 infection (Figure 8B). Likewise, the anti-FHV-1 effect induced by miR-26a was also suppressed in SOCS5-silenced cells (Figure 8B). Therefore, these data further proved that miR-26a enhanced p-STAT1 expression level via targeting SOCS5, and SOCS5 is a key factor in miR-26a-mediated anti-FHV-1.

## 4. Discussion

In this study, we tried to explore some miRNAs involved in the FHV-1 life cycle through deep sequencing. Among these differentially expressed miRNAs after FHV-1 infection, a miRNA attracted our attention because the GO analysis indicated that the miRNA, miR-26a, may participate in the IFN-related pathway. We found that (i) miR-26a can suppress FHV-1 replication; (ii) miR-26a can significantly enhance IFN-I expression levels or IFN-I-induced signalling cascades by directly targeting SOCS5, a negative regulator of the JAK-STAT signalling pathway; and (iii) miR-26a was significantly increased after FHV-1 infection in a cGAS-dependent pathway, while SOCS5 was markedly decreased. All of these data demonstrate that miR-26a plays an important role in host defence against FHV-1 infection.

To date, eight SOCS family proteins have been found in mammals, including SOCS1-7 and CIS [31], which share a conserved structural domain consisting of an N-terminal region of variable length and sequence, a central Src Homology 2 (SH2) domain and a C-terminal SOCS box motif [32]. This family mainly functions in negatively regulating the JAK-STAT pathway [33,34], which is essential for the activation of downstream antiviral ISGs [35]. Herpes simplex virus type 1 (HSV-1) induces both SOCS1 [36] and SOCS3 [37,38] to block type I IFN at early infection stage within 6 h. HSV-1 infection of HEL-30 keratinocytes promoted the SOCS-1 promoter and increased its transcription [39]. Within 6 h post-infection, HSV-1 induced both SOCS1/3 expression, which inhibited the phosphorylation of STAT1 [38,39]. However, while SOCS1 and SOCS3 were induced after HSV-1 infection, another member of SOCS family, CIS, was downregulated in these cells. In this study, we found that FHV-1 infection decreased the expression of SOCS5.

SOCS5 is a member of the SOCS family and is able to bind with the JAK kinase domain to block the phosphorylation of JAK1 and JAK2 and, therefore, further inhibit the phosphorylation of STAT1 and STAT2 [40]. In this study, overexpression of SOCS5 can inhibit IFN-induced signalling and promote FHV-1 replication, while knockdown of endogenous SOCS5 enhances the IFN-I signalling cascade and suppresses FHV-1 replication (Figure 7), which further verified the negative regulatory role of SOCS5. Previous studies have reported that some miRNAs are involved in the innate immune response by targeting SOCS family proteins [22,23,24]. We found that miR-26a can directly target SOCS5 and decrease its expression and promote the STAT1 phosphorylation, which is used to inhibit viral replication by the host. Because SOCS5 showed a significant decrease after FHV-1 infection and displayed a negative correlation with the kinetic expression profiles of miR-26a (Figure 4F,I). But we cannot ensure that the downregulation of SOCS5 is entirely regulated by miR-26a, since maybe the regulation of SOCS5 is a very complex process related to many other signalling pathways. Nevertheless, the existing experiment results are enough to verify that miR-26a really targets and influences the expression of SOCS5 to a certain extent.

The IFN system is an important component in the process of the host antiviral response toa virus, which mainly functions by stimulating the expression of various downstream ISGs, such as ISG15, MX-1, IFITM1, and OASL, among others [29]. miRNAs, as new regulators in the host, have been illustrated to play vital regulatory roles in the antiviral process [41,42,43], including the crucial antiviral defender type I interferon signalling pathway [44]. Many research results have reported that some miRNAs are involved in viral life cycles by regulating the innate immune response. For example, miR-146a facilitates replication of dengue virus by dampening interferon induction by targeting TRAF6 [45], and miR-155 enhances type I interferon expression to suppress infectious burse disease virus (IBDV) replication via targeting negative regulators, SOCS1 and TANK [46]. Additionally, miRNAs can regulate downstream IFN-I-induced signalling. miR-29a can facilitate replication of the respiratory syncytial virus (RSV) by targeting IFNAR1 [47], and miR-373 promotes HSV-1 replication through suppression of the type I IFN response by targeting the ISG IRF1 [48]. All of these miRNAs and interferon pathway components form a complex and broader regulatory network.

On the other hand, herpesviruses inhibit the early innate immune response in many different ways, and this feature is important for the establishment of infection. HSV-1 infection activates a host type I IFN response in the early infection, but the functionality of this response is subsequently blocked by viral proteins [49,50], including ICP0 [51], US11 [52], UL36 [53], UL42 [54], VP16 [55], VP24 [56], US3 [57] and UL46 [58]. Our research group reported that the IFN pathway was activated upon FHV-1 infection through the cGAS pathway at early infection but was soon blocked by multi-ORFs of FHV-1 [13]. However, few reports have revealed how hosts activate various antiviral defences to inhibit viral immune evading following infection. cGAS, as an important sensor, could detect DNA virus entry. In this study, we found that miR-26a was an antiviral factor that was induced through the cGAS pathway. Additionally, the host can detect FHV-1 entry through the cGAS pathway and then upregulates miR-26a to inhibit viral replication. This study is the first to report that cGAS mediates induction of miR-26a in herpesvirus infection. However, further exploration is required to investigate the precise mechanism(s).

In this study, we found that miR-26a induced the STAT1 phosphorylation during FHV-1 infection, which also increased the IFN-β expression. Knockdown of the IFNAR1 did not inhibit miR-26a induced STAT1 phosphorylation upon FHV-1 infection (Figure 6D), but knockdown of the SOCS5 blocked miR-26a induced STAT1 phosphorylation upon FHV-1 infection (Figure 8A). So, both results demonstrated that FHV-1 infection induced the miR-26a-SOCS5-STAT1-IFN axis to inhibit viral replication.

In summary, our present study revealed that miR-26a enhanced IFN-I antiviral signalling and suppressed FHV-1 infection by targeting SOCS5, a negative regulator of the JAK-STAT signalling pathway. These findings enrich the network constructed by both innate immune pathways and host miRNAs and provide new insight into the roles of miRNAs in host defence against viral infections.

## Supplementary Materials

The following are available online at https://www.mdpi.com/1999-4915/12/1/2/s1. Table S1: Sequences of miRNA mimics, inhibitors and siRNAs, Table S2: Table S2. Sequences of primers used in the research; Table S3: Table S3. Some differentially expressed miRNAs obtained from the high-throughput sequencing.

## References

[1] Davison A.J., Eberle R., Ehlers B., Hayward G.S., McGeoch D.J., Minson A.C., Pellett P.E., Roizman B., Studdert M.J., Thiry E. (2009). The order Herpesvirales. Arch. Virol..

[2] Stanley J., Bisaro D., Briddon R., Brown J., Fauquet C., Harrison B., Rybicki E., Stenger D.C. (2005). Geminivridae: Eighth Report of the ICTV on Virus Taxonomy. Virus Taxonomy-Eighth Report of the International Committee on Taxonomy of Viruses.

[3] Daniels M.J., Golder M.C., Jarrett O., MacDonald D.W. (1999). MacDonald, Feline viruses in wildcats from Scotland. J. Wildl. Dis..

[4] Hofmann-Lehmann R., Fehr D., Grob M., Elgizoli M., Packer C., Martenson J.S., O’Brien S.J., Lutz H. (1996). Prevalence of antibodies to feline parvovirus, calicivirus, herpesvirus, coronavirus, and immunodeficiency virus and of feline leukemia virus antigen and the interrelationship of these viral infections in free-ranging lions in east Africa. Clin. Diagn. Lab. Immunol..

[5] Munson L., Wack R., Duncan M., Montali R.J., Boon D., Stalis I., Crawshaw G.J., Cameron K.N., Mortenson J., Citino S. (2004). Chronic eosinophilic dermatitis associated with persistent feline herpes virus infection in cheetahs (Acinonyx jubatus). Vet. Pathol..

[6] Ostrowski S., van Vuuren M., Lenain D.M., Durand A. (2003). A serologic survey of wild felids from central west Saudi Arabia. J. Wildl. Dis..

[7] Paul-Murphy J., Work T., Hunter D., McFie E., Fjelline D. (1994). Serologic survey and serum biochemical reference ranges of the free-ranging mountain lion (Felis concolor) in California. J. Wildl. Dis..

[8] Van Vuuren M., Goosen T., Rogers P. (1999). Feline herpesvirus infection in a group of semi-captive cheetahs. J. S. Afr. Vet. Assoc..

[9] Thiry E., Addie D., Belák S., Boucraut-Baralon C., Egberink H., Frymus T., Gruffydd-Jones T., Hartmann K., Hosie M.J., Lloret A. (2009). Feline herpesvirus infection. ABCD guidelines on prevention and management. J. Feline Med..

[10] Townsend W.M., Jacobi S., Tai S.H., Kiupel M., Wise A.G., Maes R.K. (2013). Ocular and neural distribution of feline herpesvirus-1 during active and latent experimental infection in cats. BMC Vet. Res..

[11] Albà M.M., Das R., Orengo C.A., Kellam P. (2001). Genomewide function conservation and phylogeny in the Herpesviridae. Genome Res..

[12] Tai S.S., Niikura M., Cheng H.H., Kruger J.M., Wise A.G., Maes R.K. (2010). Complete genomic sequence and an infectious BAC clone of feline herpesvirus-1 (FHV-1). Virology.

[13] Tian J., Liu Y., Liu X., Sun X., Zhang J., Qu L. (2018). Feline Herpesvirus 1 US3 Blocks the Type I Interferon Signal Pathway by Targeting Interferon Regulatory Factor 3 Dimerization in a Kinase-Independent Manner. J. Virol..

[14] Kloosterman W.P., Plasterk R.H. (2006). The diverse functions of microRNAs in animal development and disease. Dev. Cell.

[15] Bushati N., Cohen S.M. (2007). microRNA functions. Annu. Rev. Cell Dev. Biol..

[16] Wilson R.C., Doudna J.A. (2013). Molecular mechanisms of RNA interference. Annu. Rev. Biophys..

[17] Kim Y.K., Heo I., Kim V.N. (2010). Modifications of small RNAs and their associated proteins. Cell.

[18] Bartel D.P. (2004). MicroRNAs: Genomics, biogenesis, mechanism, and function. Cell.

[19] Zhu B., Ye J., Nie Y., Ashraf U., Zohaib A., Duan X., Fu Z.F., Song Y., Chen H., Cao S. (2015). MicroRNA-15b Modulates Japanese Encephalitis Virus-Mediated Inflammation via Targeting RNF125. J. Immunol..

[20] Yin Y., Liu W., Dai Y. (2015). SOCS3 and its role in associated diseases. Hum. Immunol..

[21] Wang P., Hou J., Lin L., Wang C., Liu X., Li D., Ma F., Wang Z., Cao X. (2010). Inducible microRNA-155 feedback promotes type I IFN signaling in antiviral innate immunity by targeting suppressor of cytokine signaling 1. J. Immunol..

[22] Ma Y., Wang C., Xue M., Fu F., Zhang X., Li L., Yin L., Xu W., Feng L., Liu P. (2018). The Coronavirus Transmissible Gastroenteritis Virus Evades the Type I Interferon Response through IRE1alpha-Mediated Manipulation of the MicroRNA miR-30a-5p/SOCS1/3 Axis. J. Virol..

[23] Fu M., Wang B., Chen X., He Z., Wang Y., Li X., Cao H., Zheng S.J. (2018). MicroRNA gga-miR-130b Suppresses Infectious Bursal Disease Virus Replication via Targeting of the Viral Genome and Cellular Suppressors of Cytokine Signaling 5. J. Virol..

[24] Sharma N., Kumawat K.L., Rastogi M., Basu A., Singh S.K. (2016). Japanese Encephalitis Virus exploits the microRNA-432 to regulate the expression of Suppressor of Cytokine Signaling (SOCS) 5. Sci. Rep..

[25] Haldipur B., Bhukya P.L., Arankalle V., Lole K. (2018). Positive Regulation of Hepatitis E Virus Replication by MicroRNA-122. J. Virol..

[26] Zhang Q., Guo X.K., Gao L., Huang C., Li N., Jia X., Liu W., Feng W.H. (2014). MicroRNA-23 inhibits PRRSV replication by directly targeting PRRSV RNA and possibly by upregulating type I interferons. Virology.

[27] Zhang J., Li Z. (2020). Identification and Analysis of the Profile of Viral and Host microRNAs in Feline Herpesvirus 1-Infected CRFK cells. Virol. J..

[28] Sun J.Z., Wang J., Wang S., Yuan D., Birame B.M., Li Z., Yi B., Liu W. (2014). MicroRNA profile analysis of a feline kidney cell line before and after infection with mink enteritis virus. Gene.

[29] Schneider W.M., Chevillotte M.D., Rice C.M. (2014). Interferon-stimulated genes: A complex web of host defenses. Annu. Rev. Immunol..

[30] Sun L., Wu J., Du F., Chen X., Chen Z.J. (2013). Cyclic GMP-AMP synthase is a cytosolic DNA sensor that activates the type I interferon pathway. Science.

[31] Linossi E.M., Calleja D.J., Nicholson S.E. (2018). Understanding SOCS protein specificity. Growth Factors.

[32] Hilton D.J., Richardson R.T., Alexander W.S., Viney E.M., Willson T.A., Sprigg N.S., Starr R., Nicholson S.E., Metcalf D., Nicola N.A. (1998). Twenty proteins containing a C-terminal SOCS box form five structural classes. Proc. Natl. Acad. Sci. USA.

[33] Yoshimura A., Ito M., Chikuma S., Akanuma T., Nakatsukasa H. (2018). Negative Regulation of Cytokine Signaling in Immunity. Cold Spring Harb. Perspect. Biol..

[34] Linossi E.M., Nicholson S.E. (2015). Kinase inhibition, competitive binding and proteasomal degradation: Resolving the molecular function of the suppressor of cytokine signaling (SOCS) proteins. Immunol. Rev..

[35] Lin R.J., Chang B.L., Yu H.P., Liao C.L., Lin Y.L. (2006). Blocking of interferon-induced Jak-Stat signaling by Japanese encephalitis virus NS5 through a protein tyrosine phosphatase-mediated mechanism. J. Virol..

[36] Sato Y., Koshizuka T., Ishibashi K., Hashimoto K., Ishioka K., Ikuta K., Yokota S.I., Fujii N., Suzutani T. (2017). Involvement of herpes simplex virus type 1 UL13 protein kinase in induction of SOCS genes, the negative regulators of cytokine signaling. Microbiol. Immunol..

[37] Yokota S., Yokosawa N., Okabayashi T., Suzutani T., Fujii N. (2005). Induction of suppressor of cytokine signaling-3 by herpes simplex virus type 1 confers efficient viral replication. Virology.

[38] Yokota S., Yokosawa N., Okabayashi T., Suzutani T., Miura S., Jimbow K., Fujii N. (2004). Induction of suppressor of cytokine signaling-3 by herpes simplex virus type 1 contributes to inhibition of the interferon signaling pathway. J. Virol..

[39] Frey K.G., Ahmed C.M., Dabelic R., Jager L.D., Noon-Song E.N., Haider S.M., Johnson H.M., Bigley N.J. (2009). HSV-1-induced SOCS-1 expression in keratinocytes: Use of a SOCS-1 antagonist to block a novel mechanism of viral immune evasion. J. Immunol..

[40] Linossi E.M., Chandrashekaran I.R., Kolesnik T.B., Murphy J.M., Webb A.I., Willson T.A., Kedzierski L., Bullock A.N., Babon J.J., Norton R.S. (2013). Suppressor of Cytokine Signaling (SOCS) 5 utilises distinct domains for regulation of JAK1 and interaction with the adaptor protein Shc-1. PLoS ONE.

[41] Bruscella P., Bottini S., Baudesson C., Pawlotsky J.M., Feray C., Trabucchi M. (2017). Viruses and miRNAs: More Friends than Foes. Front. Microbiol..

[42] Piedade D., Azevedo-Pereira J.M. (2016). The Role of microRNAs in the Pathogenesis of Herpesvirus Infection. Viruses.

[43] Frappier L. (2015). Regulation of herpesvirus reactivation by host microRNAs. J. Virol..

[44] Forster S.C., Tate M.D., Hertzog P.J. (2015). MicroRNA as Type I Interferon-Regulated Transcripts and Modulators of the Innate Immune Response. Front. Immunol..

[45] Wu S., He L., Li Y., Wang T., Feng L., Jiang L., Zhang P., Huang X. (2013). miR-146a facilitates replication of dengue virus by dampening interferon induction by targeting TRAF6. J. Infect..

[46] Wang B., Fu M., Liu Y., Wang Y., Li X., Cao H., Zheng S.J. (2018). gga-miR-155 Enhances Type I Interferon Expression and Suppresses Infectious Burse Disease Virus Replication via Targeting SOCS1 and TANK. Front. Cell. Infect. Microbiol..

[47] Zhang Y., Yang L., Wang H., Zhang G., Sun X. (2016). Respiratory syncytial virus non-structural protein 1 facilitates virus replication through miR-29a-mediated inhibition of interferon-alpha receptor. Biochem. Biophys. Res. Commun..

[48] Xie Y., He S., Wang J. (2018). MicroRNA-373 facilitates HSV-1 replication through suppression of type I IFN response by targeting IRF1. Biomed. Pharmacother..

[49] Zheng C. (2018). Evasion of Cytosolic DNA-Stimulated Innate Immune Responses by Herpes Simplex Virus 1. J. Virol..

[50] Melroe G.T., DeLuca N.A., Knipe D.M. (2004). Herpes simplex virus 1 has multiple mechanisms for blocking virus-induced interferon production. J. Virol..

[51] Lin R., Noyce R.S., Collins S.E., Everett R.D., Mossman K.L. (2004). The herpes simplex virus ICP0 RING finger domain inhibits IRF3- and IRF7-mediated activation of interferon-stimulated genes. J. Virol..

[52] Xing J., Wang S., Lin R., Mossman K.L., Zheng C. (2012). Herpes simplex virus 1 tegument protein US11 downmodulates the RLR signaling pathway via direct interaction with RIG-I and MDA-5. J. Virol..

[53] Wang S., Wang K., Li J., Zheng C. (2013). Herpes simplex virus 1 ubiquitin-specific protease UL36 inhibits beta interferon production by deubiquitinating TRAF3. J. Virol..

[54] Zhang J., Wang S., Wang K., Zheng C. (2013). Herpes simplex virus 1 DNA polymerase processivity factor UL42 inhibits TNF-alpha-induced NF-kappaB activation by interacting with p65/RelA and p50/NF-kappaB1. Med. Microbiol. Immunol..

[55] Xing J., Ni L., Wang S., Wang K., Lin R., Zheng C. (2013). Herpes simplex virus 1-encoded tegument protein VP16 abrogates the production of beta interferon (IFN) by inhibiting NF-kappaB activation and blocking IFN regulatory factor 3 to recruit its coactivator CBP. J. Virol..

[56] Zhang D., Su C., Zheng C. (2016). Herpes Simplex Virus 1 Serine Protease VP24 Blocks the DNA-Sensing Signal Pathway by Abrogating Activation of Interferon Regulatory Factor 3. J. Virol..

[57] Wang S., Wang K., Lin R., Zheng C. (2013). Herpes simplex virus 1 serine/threonine kinase US3 hyperphosphorylates IRF3 and inhibits beta interferon production. J. Virol..

[58] You H., Zheng S., Huang Z., Lin Y., Shen Q., Zheng C. (2019). Herpes Simplex Virus 1 Tegument Protein UL46 Inhibits TANK-Binding Kinase 1-Mediated Signaling. MBio.

