# Supplementary material for: miR-26a Inhibits Feline Herpesvirus 1 Replication by Targeting SOCS5 and Promoting Type I Interferon Signaling
Source: Viruses. 2019 Dec 18;12(1):2. doi: 10.3390/v12010002 (PMC7020096; doi:10.3390/v12010002)
Supplement: Supplementary file 1 [file viruses-12-00002-s001.pdf]

Table S1. Sequences of miRNA mimics, inhibitors and siRNAs.

miRNAs/siRNAs	Sequences (5′-3′)	
	sense (5′-3′)	antisense (5′-3′)
miR-26a mimics	TTCAAGTAATCCAGGATAGGCT	CCUAUCCUGGAUUACUUGAAUU
miR-26a inhibitors	AGCCTATCCTGGATTACTTGAA	
sicGAS	UCGGGACCAAAUUGACAAATT	UUUGUCAAUUUGGUCCCGUTT
siIFNAR1#1	GTGCCTATAGTCCAGTGTA	
siIFNAR1#2	CTCCAACCATTAACATGAA	
siIFNAR1#3	GGATTTGTGCTGCCTTATT	
siSOCS5#1	GGAACAAGACUGGCAAGAATT	UUCUUGCCAGUCUUGUUCCTT
siSOCS5#2	GCACAGGUUAAUCCGUUAUTT	AUAACGGAUUAACCUGUGCTT
siSOCS5#3	GGAAUUGAUGGGCUCCCUUTT	AAGGGAGCCCAUCAAUUCCTT

Table S2. Sequences of primers used in the research.

Primers	Sequences (5′-3′)
RT-miR-26a	GTCGTATCCAGTGCAGGGTCCGAGGTATTCGCACTGGATAC GACAGCCTATC
RT-miR-101	GTCGTATCCAGTGCAGGGTCCGAGGTATTCGCACTGGATAC GACCTTCAGTT
miR-26a-F	CGGGGCTTCAAGTAATCCAG
miR-101-F	TACAGTACTGTGATAACTGAAG
U6-F	CTCGCTTCGGCAGCACA
U6-R	AACGCTTCACGAATTTGCGT
miR-UR-R	CAGTGCAGGGTCCGAGGTAT
qISG15-F	TCCTGGTGAGGAACCACAAGGG
qISG15-R	TTCAGCCAGAACAGGTCGTC
qViperin-F	CATGACCGGGGCGAGTACCTG
qViperin-R	GCAAGGATGTCCAAATATTCACC
qIFITM1-F	CACCACCGTGATCAACATCCA
qIFITM1-R	GACTTCACGGAGTAGGCAAAG
qSOCS5-F	TGTGTCACCCCAGGAACAAG
qSOCS5-R	CTTACGCCATACCGCCTCTC
qIFN-β-F	GAAGGAGGAAGCCATATTGGT
qIFN-β-R	CTCCATGATTTCCTCCAGGAT
q18S-F	CGGCTACCACATCCAAGGAA
q18S-R	GCTGGAATTACCGCGGCT
miR-26-socs5-3'UTR (WT)-F	TCTAGTTGTTTAAACGAGCTCTTTGGATGGCAGTATTTATATC TTTGT
miR-26-socs5-3'UTR (WT)-R	CAGGTCGACTCTAGACTCGAGTACACACAAACTGGAACAT GTGACTT
miR-26-socs5-3'UTR (mut)-R1	CAAAATAGCTCACGACCTGCGTATTGCATA
miR-26-socs5-3'UTR (mut)-F2	GCAGGTCGTGAGCTATTTTGGAAGTAA

Table S3. Some differentially expressed miRNAs obtained from the high-throughput sequencing.

miRNA id	FHV-1[a]	Mock[a]	log2 Ratio (FHV-1/Mock)	Up/Down	p-value
miR-146-5p	150	1242	-4.350897495	Down	0
miR-30a-5p	1282	10596	-3.289424101	Down	0
miR-200a-3p	161	1263	-3.214380608	Down	0
miR-378	1009	5671	-2.733087788	Down	0
miR-196b	1802	9489	-2.639084641	Down	0
miR-199a-3p	900	3602	-2.243278191	Down	5.79E-255
miR-20-5p	1090	4220	-2.195170953	Down	6.09E-57
miR-339b-5p	1950	6930	-2.072303034	Down	7.24E-86
miR-27a-5p	9750	29816	-1.855005293	Down	0
miR-196a	16114	48944	-1.845203685	Down	0
miR-125b-1-3p	485	1259	-1.618745357	Down	2.75E-110
miR-27b-5p	877	2062	-1.475717739	Down	0
miR-125a-3p	604	1322	-1.372540182	Down	8.62E-91
miR-1187	1419	483	1.312378631	Up	2.72E-75
miR-374a-5p	689	231	1.334604539	Up	2.35E-38
miR-155-5p	14705	4770	1.381837317	Up	0
miR-181a-3p	356	158	1.415037499	Up	0
miR-222a-5p	663	253	1.389871485	Up	3.80E-24
miR-25-3p	24283	7787	1.398407229	Up	7.85E-27
miR-23b-5p	6150	2317	1.408328366	Up	8.41E-71
miR-574-5p	4931	1748	1.496175068	Up	0
miR-26a-5p	97127	19293	2.574178054	Up	0
miR-101	5753	888	2.69568288	UP	0
miR-17-5p	1423	128	3.231834629	Up	0

[a] Mean reads from triple samples
